# Ant Community Is Not Influenced by the Addition of Olive Mill Pomace Compost in Two Different Olive Crop Managements

**DOI:** 10.3390/insects14100783

**Published:** 2023-09-26

**Authors:** José E. González-Zamora, José M. Gamero-Monge, Rosa Pérez-de la Luz

**Affiliations:** Departamento de Agronomía, Universidad de Sevilla, 41013 Sevilla, Spain; jisaro@hotmail.es (J.M.G.-M.); rosaperezdelaluz@gmail.com (R.P.-d.l.L.)

**Keywords:** alperujo, superhigh density, tillage, cover crop, *Pheidole pallidula*, *Plagiolepis* spp.

## Abstract

**Simple Summary:**

Olive oil production is the main economic interest in olive orchards, but this industrial process generates large amounts of a by-product called ‘alperujo’, which is very negative to the environment. When composted, it generates a product useful for fertilizing many crops, and the objective of the work was to evaluate its impact on the ant community in two diverse types of olive crop management: a superintensive grove (no-tillage, with a cover crop, irrigated) and a traditional grove (tillage, no cover crop, rain-fed). The main conclusion was that the addition of compost did not alter the presence of ants and the composition of the species in each grove, but the type of management could have influenced the abundance of ants (much more abundant in the superintensive than in the traditional grove) and, secondarily, the composition of the species.

**Abstract:**

Industrial production of olive oil generates large amounts of solid waste called ‘alperujo’. Its compost can fertilize many crops, especially olives. Furthermore, superintensive orchards are increasing their surface globally due to higher production and savings in different costs. Ants are considered an important part of the arthropod community in olive orchards and could even play a significant role in pest control. The tree canopy and ground were sampled to compare the ant assemblage in plots fertilized with compost and mineral products in two groves with different types of crop management (superintensive and traditional) over two years. The numbers of ants in both types of fertilization in each grove were not statistically different (*p* > 0.05), indicating that the type of fertilization did not have a significant impact on its populations in the ground or in the canopy, but the number of individuals was significantly higher in the superintensive grove than in the traditional grove (both in the ground and in the canopy, *p* < 0.01). The most frequent species in the ground were *Pheidole pallidula*, *Plagiolepis smitzii* and *Aphaenogaster senilis* (superintensive grove) and *Pheidole pallidula*, *Tetramorium* gr *semilaeve*, *Plagiolepis pygmaea*, and *Tapinoma nigerrimum* (traditional grove). In the canopy, the most frequent species were *Plagiolepis* spp. in both groves. Differences in ant densities and species between the groves could be due to the different management, especially of the soil, but it must be confirmed using more replicas and longer periods of study.

## 1. Introduction

Olives are the most important fruit crop in Spain, with 2.76 × 10^6^ ha, most of it (around 2.55 × 10^6^ ha) dedicated to obtaining oil from the drupe [1]. Production of olive oil generates a solid pomace waste denominated in Spanish as ‘alperujo’ in great quantities (500–800 kg per tonne of processed olives have been reported [2,3]). This ‘alperujo’ has different chemical properties making it an environmentally hazardous product, and difficult to manage [2]. Composting this by-product and using it as a fertilizer can generate savings in mineral fertilization and other benefits [4], helping to reduce the use of inorganic fertilizers, reducing its impact on the environment, and contributing to a circular economy of resources and sustainable agriculture [5].

Olive agriculture is also changing in management, especially in Spain and in areas with irrigation, more or less leveled and with high production potential, where new orchards with superintensive management (superhigh densities of trees are planted in hedges to form continuous vegetation) are gaining interest and area, mainly due to the higher olive yield and the savings in harvesting costs and other cultural operations [6,7]. The increase in the area of irrigated olive dedicated to oil production in Spain (mostly in superintensive management) is about 54% in the last years (366,410 ha in 2009 to 562,980 ha in 2022) [1,8].

The addition of compost (or other by-products of agriculture, such as manure and sewage) has significant effects on the crop because it incorporates different nutrients and carbon in the soil and can influence the soil’s inhabitants. This latter aspect is the subject of different studies [9,10,11,12,13] with extensive reviews, which in general highlight the beneficial effect on the soil fauna, with an increase in arthropods such as Acari (order Oribatida as the most mentioned, but also Mesostigmata and Prostigmata), Collembola, some families of Coleoptera (Carabidae it is one of the most cited), and other inhabitants such as Nematoda or Lumbricidae (earthworms), but depending on the type of by-product used and the specific conditions (crop, management) of the study.

Other studies have been carried out on the olive crop in Spain (but also in other countries with extensive olive areas) to analyze the effect of different variables (such as crop and soil management from a broad point of view, landscape complexity, and others) on the diversity and complexity of soil and canopy inhabitants to maximize and strengthen resilience [14,15,16,17,18], natural biological control of some olive pests [19,20,21,22,23,24,25] or improve the presence of some particular natural enemies [26,27]. A general common result of these studies indicates that higher landscape complexity [17,18], organic management [19], the use of cover crops [16], and no-tillage [14,15] help to increase biodiversity, which ultimately can help to improve the control of some olive pests; the most important and cited are *Prays oleae* Bernard (Lepidoptera: Praydidae) [21,22,24] and *Bactrocera oleae* (Rossi) (Diptera, Tephritidae) [19,20]. On the contrary, intensive management (with soil tillage, generally no cover crop, and intensive use of inputs) generally reduces this diversity and the potential for biological control of different olive pests [23,25].

Ants are one of the most abundant groups of arthropods in the world, with rather stable populations and feeding habits that have a major influence on many habitats [28]. They are of remarkable interest in olive orchards [14,29,30,31,32,33,34] and are considered an important factor of diversity in soil communities that can even help to limit populations of certain olive pests, such as *P. oleae* [35,36,37]. Ant species composition in olive groves has been the objective of several studies [29,30,33,37,38] or included in a more general view [14,18,34].

Few works (such as Gkisakis et al. [14]) have studied the effect of compost/manure on the ant assemblage on crops, including olive groves. In addition, incorporating a superintensive type of olive orchard in the design of experiments is not very common in this type of study, but it is gaining importance in specific areas of Spain and the world [1,8]. The present work focuses on the effect of the addition of ‘alperujo’ compost on the ant assemblage compared to the use of mineral fertilization. The main hypothesis is that ‘alperujo’ compost can modify different aspects of the soil where it is applied, varying the ant numbers and/or species composition. Compost can influence the mesofauna in the soil, promoting arthropods that can be prey to ants, or compete with them; but compost also can alter physical and chemical properties of the soil (not specifically studied in this work) that could influence the ant assemblage both in soil and tree canopy. The study is carried out in two models of olive layout and management: superintensive and traditional, but with a limited number of replications. The main results obtained in this work are that the addition of compost did not influence the number of ants and species composition within each grove, but the type of management was important, with more abundance of ants in the superintensive model than in the traditional model, probably due to differential soil management.

## 2. Materials and Methods

### 2.1. Location

**Figure A1:**
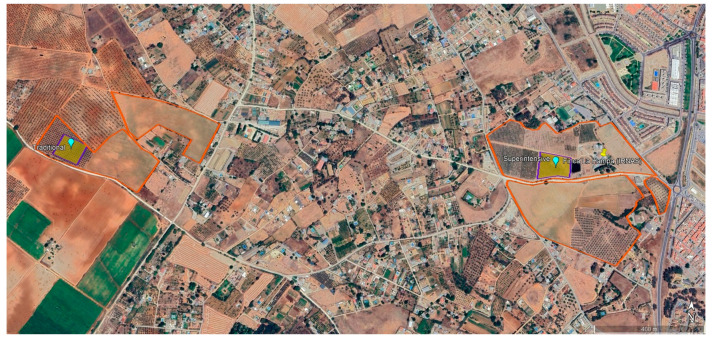
General location of the Hampa farm (outlined in orange), with the two groves (Superintensive and Traditional, outlined in blue and filled with yellow) where the research was conducted.

This research was carried out in the experimental farm “La Hampa” located in Coria del Río (Seville, Spain) (37°17.010′ N 6°3.936′ W), belonging to the Institute of Natural Resources and Agrobiology of Seville (Instituto de Recursos Naturales y Agrobiología de Sevilla, Seville, Spain), at an average of 20 m a.s.l. The climate is typically Mediterranean, with mild rainy winters and very hot, dry summers (with an average temperature in July and August of 25.9 and 25.4 °C, for 2021 and 27.9 and 25.3 °C for 2022, respectively (data from the in situ meteorological station). The experimental farm is surrounded by a mosaic of olive and stone fruit orchards, arable land (dedicated to cereals, cotton, and sunflower), patches of pine woods, and second homes and small farmhouses. The research was conducted in two experimental groves of the farm: a superintensive olive grove and a traditional olive grove (Appendix A, Figure A1).

### 2.2. Experimental Design

**Figure A2:**
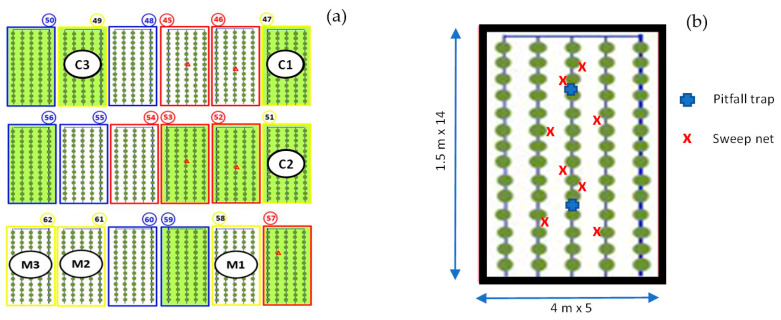
Superintensive grove: (**a**) location of the plots (C, Compost; M, Mineral); (**b**) location of the pit fall traps and where the sweep net was used in the two central alleys of each plot.

The superintensive olive grove was planted in 2018 with the olive cv ‘Manzanilla de Sevilla’, using a planting layout of 4 m × 1.5 m (a superhigh density of 1667 trees per ha) that forms a hedge. It was divided into 18 elemental plots of 20 m × 21 m (ca. 420 m^2^), each comprising five rows of trees of around 21 m in length with 70 trees per plot (Appendix A, Figure A2). Six plots were used in this research; three of them were fertilized with composted residues of olive mill pomace (treatment named ‘Compost’) originated from the oil extraction of the olive fruit, while the other three plots were fertilized with mineral products (treatment named ‘Mineral’, see Appendix A, Table A1), and the six plots were randomly distributed in the grove. Both treatments (Compost and Mineral) were foliar fertilized with the same products and schedule, and drip irrigated after a regulated deficit irrigation procedure [39], which limited the average annual water irrigation received for each treatment during this study to 360.4 ± 27.7 mm (Compost) and 308.1 ± 8.7 mm (Mineral) in 2021 and 197.0 ± 14.6 mm (Compost) 203.1 ± 3.5 mm (Mineral) in 2022. During the first year of the study (2021), the application of compost was conducted for the first time in the grove in July (17 t·ha^−1^), and the mineral fertilization was limited only to foliar fertilization in both treatments, but no fertigation was conducted in the mineral treatment, and in 2022, compost was added in March, (17 t·ha^−1^) and the mineral treatment was fertigated (see Appendix A, Table A1). This grove kept a permanent cover crop (mainly Poaceae) in the tree rows and alleys, no tillage was conducted in the two years of the study, and only the cover was mowed in May–June in both years. During this study, different pesticides were applied (Appendix A, Table A2), following the integrated pest management recommendations of the regional government.

**Figure A3:**
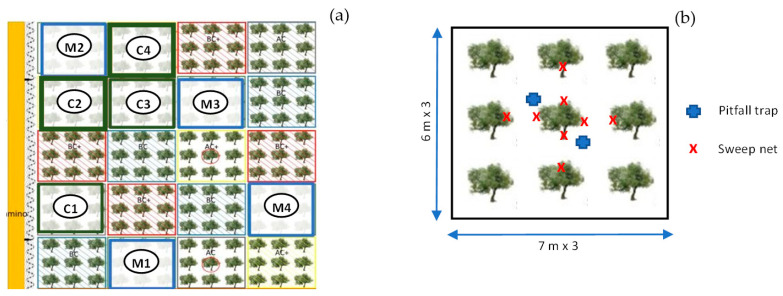
Traditional grove: (**a**) location of the plots (C, Compost; M, Mineral); (**b**) location of the pit fall traps and where the sweep net was used in each plot.

The traditionally managed grove was planted in 1998 with the olive cv ‘Manzanilla de Sevilla’, following a traditional olive grove design of 7 m × 6 m (238 trees per ha) and trees formed on a single foot with two main branches. It was divided into 20 plots of 21 m × 18 m (ca 380 m^2^), with nine trees per plot (Appendix A, Figure A3). In this investigation eight plots were used, four of them were fertilized with composted residues from olive mill pomace (treatment named ‘Compost’), while the other four plots were fertilized with mineral products (treatment named ‘Mineral’, see Appendix A, Table A1), and the eight plots were randomly distributed within the grove. The addition of olive mill compost started in 2018 and was conducted in 2020 (December, with 17 t·ha^−1^) and 2022 (March, with 17 t·ha^−1^), every two years, while mineral fertilization was not applied in 2021, but in 2022 (see Appendix A, Table A1). The grove is rain-fed (366.1 mm in the agronomic year from October 2020 to September 2021 and 303.3 mm from October 2021 to September 2022) and managed more traditionally, with soil tillage twice a year (January and July). During this study, different pesticides were applied (Appendix A, Table A2), following the integrated pest management recommendations of the regional government.

### 2.3. Sampling

Three sampling methods were used to study the influence of the type of fertilization in the ant assemblage in both olive groves [40]. Visual sampling was conducted to obtain information directly on shoots and inflorescences and possible relations between ants and other arthropods, but it was not included because the numbers observed were extremely low. Sweep net sampling complements visual sampling because it can obtain individuals not easily observed in the canopy (as for example in [31,36]) and, besides, it is a quantitative method of sampling. Finally, pitfall traps are necessary to study the diversity of ants at ground level, where most ants live, and could show a stronger effect due to the type of fertilizer used [14,30,33,41].

Sweep net sampling was conducted using a net of 47 cm in diameter with a 42 cm handle. The net was used by sweeping the branches and collecting (after finishing the procedure in each plot) with an aspirator into a vial of all the arthropods captured inside the net. The sampling was conducted as soon as possible in the morning. The vials were labeled and kept in cool conditions (in a cardboard box with freezer blocks) until they reached the lab, where the vials were placed in a −18 °C freezer for 24 h, and then the specimens were determined to order and family/species, if possible, with the aid of a stereomicroscope (×45) and different taxonomic keys [42,43]. In the superintensive grove, the sampling was conducted by sweeping the branches with the sweep net four times while walking along each of the two central alleys of the plot (a total of eight sweeps were conducted in each plot) (Appendix A, Figure A2). In the traditional grove, the sampling was also conducted by sweeping the branches with the sweep net in the four cardinal orientations of the central tree and one sweep on the inner face of the four trees of the central cross (a total of eight sweeps were conducted in each plot) (Appendix A, Figure A3).

The pitfall trap consisted of a polyethene cup of 105 mm height and 90 mm diameter at the opening, inserted into the soil at ground level and filled with 25–30 mL of ethylene glycol: water (1:1) and covered with a plastic dish of 25 cm diameter to avoid evaporation and debris. Two traps were installed in each plot: in the central tree row and 10 m apart in the superintensive grove and the diagonal, near the central tree and around 5 m apart in the traditional grove (Appendix A, Figure A2 and Figure A3). Traps were placed in the ground for 72 h, and then the liquid with the trapped specimens was moved to a smaller cup with a lid and brought to the lab, where the specimens were evaluated as before.

For the two sampling methods, nine sampling dates were performed in 2021 (eight sampling dates for pitfall traps) and 2022 in the superintensive grove (from April until mid-October), while in the traditional grove, six sampling dates were performed in 2021 (from the end of June to mid-October) and nine in 2022 (from April until mid-October) (see Appendix A, Table A3, for the sampling dates).

Few specimens were collected in 2021 (only on three sampling dates in the Superintensive grove) and sent to specialists for species identification. In 2022, all specimens collected in eight out of nine sampling dates in both groves were identified using the Lebas et al. key [44] until the genus/group/species, when possible. Specimens collected with the sweep net were not separated by fertilization treatment, only at the level of the grove (Superintensive and Traditional) due to the low numbers and diversity observed in 2021, while the specimens collected in the pitfall traps were separated by fertilization treatment in each grove.

### 2.4. Data Analysis

Repeated measures ANOVA was used to analyze how the ant numbers (in sweep net and pitfall trap sampling) were individually affected by the fertilization treatment with a method of analysis for the time-series abundance data. With this method, it was evaluated whether fertilization treatment (between-subject effect, with two treatments, Compost and Mineral), time (within-subject effect, sampling dates), and interaction of time and fertilization treatment was significant in the response variables for each of the groves and years. The pooled data from the two years in each grove were also analyzed for each response variable, with a generalized linear model in which the binomial negative function was used to analyze the sweep net and pitfall trap sampling data (discrete data), with treatment, grove and treatment × grove as fixed factors to test whether a general pattern was present. Various species diversity indices were used to compare groves and fertilization treatments: Shannon, Simpson, Simpson inverse, and Simpson unbiased. A comparison of the frequencies of ant species (expressed as relative presence) was conducted with a Χ^2^ test of expected frequencies.

SPSS (v15.0 for Windows) was used in the repeated measures ANOVA and the Χ^2^ test of expected frequencies. R (v4.2.2) was used for the analysis of the two years together. The package ‘MASS’ (v7.3-58.3) was used to apply the binomial negative function (with the *glm.nb* function) in the sweep net and pitfall trap data. Diversity indices were performed with the package ‘Vegan’ (v2.5-2). Before applying repeated measures ANOVA, data from sweep net and pitfall trap samplings were transformed with log (x + 1).

## 3. Results

The presence of ants was studied in both the years 2021 and 2022, but special attention was taken in 2022 when the identification at the species/genera/group level was conducted in most of the individuals taken with the sweep net and pitfall traps.

The sweep net sampling collected a low number of ants (a total of 182 individuals in 2021 and 268 individuals in 2022), but higher than the visual sampling (28 observations in 2021 and 11 observations in 2022), indicating that it was a better sampling method for this group when the objective is to know the ant population in the tree canopy. A significantly higher number of ants in the canopy resulted in the superintensive grove (422 individuals) versus the traditional grove (28 individuals) in the two years analyzed together (*p* < 0.01, Table 1). When analyzing each grove, no significant difference between fertilization treatments was detected with the repeated measures analysis in 2021 (superintensive grove, *p* = 0.437; Table 1) and 2022 (superintensive grove, *p* = 0.106 and traditional grove *p* = 1.0; Table 1).

**Figure 1:**
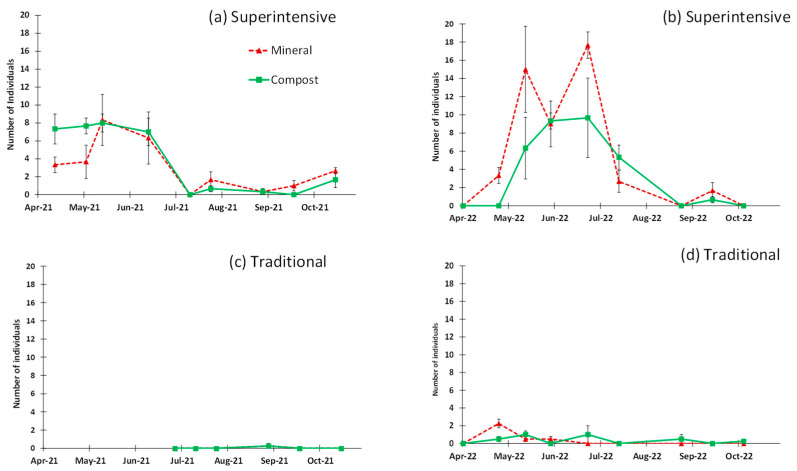
Seasonal pattern of ant captures in the canopy of the trees, obtained with the sweep net in the Superintensive grove (**a**,**b**) and in the Traditional grove (**c**,**d**); each point represents the average of captures per plot with the standard error (vertical bars).

The seasonal pattern of the ants in the canopy is of special interest in the superintensive grove (Figure 1a,b) and reflects great activity in spring, it greatly decreases with summer, but it seems to reactivate at the end of summer and the beginning of autumn. The time factor (sampling date) was very significant in the superintensive grove in both years (*p* < 0.01, Table 1), reflecting such differences during the season, but the interaction between treatment and sampling date was not significant (*p >* 0.05, Table 1). The traditional grove shows a different pattern, with a very reduced presence of ants (Figure 1c,d), without the influence of the sampling date (*p* > 0.05) or the interaction between treatment and sampling date (*p* > 0.05) in 2022 (Table 1).

Pitfall traps captured the highest numbers of ants (a total of 1417 individuals in 2021 and 1460 individuals in 2022). Again, there was a significantly higher presence of ants in the superintensive (2128 individuals) versus the traditional grove (749) in the two years analyzed together (*p* < 0.01, Table 1). Analyzing each grove, again no significant difference between fertilization treatments was obtained with the repeated measures analysis in 2021 (superintensive grove, *p* = 0.371; traditional grove *p* = 0.559; Table 1) and 2022 (superintensive grove *p* = 0.990; traditional grove *p* = 0.561; Table 1).

**Figure 2:**
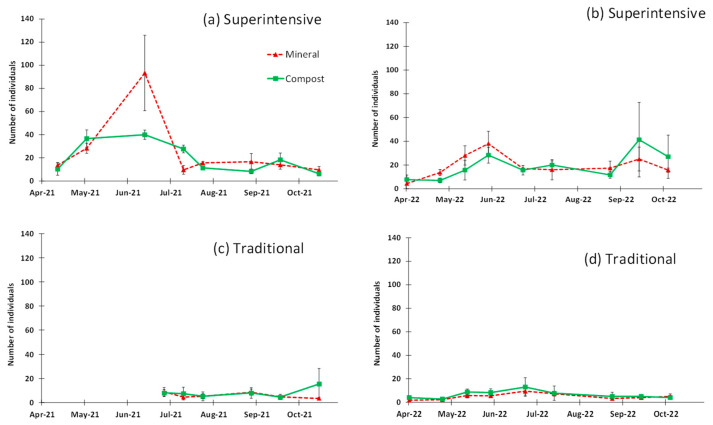
Seasonal pattern of ant captures in the ground, obtained with pitfall traps in the Superintensive grove (**a**,**b**) and in the Traditional grove (**c**,**d**); each point represents the average of captures per plot with the standard error (vertical bars).

The seasonal pattern of the ants in the soil is similar to what happened in the canopy, with greater abundance in spring and then in autumn in the superintensive grove (Figure 2a,b), but less dramatically. The time factor (sampling date) was significant in the superintensive grove in 2021 (*p* = 0.023), but not in 2022 ((*p* = 0.243) (Table 1), and the interaction between treatment and sampling date was not significant (*p >* 0.05) in any year (Table 1). In the traditional grove, there was no effect of the sampling time (*p* > 0.05) or the interaction between treatment and sampling date (*p* > 0.05) in both years (Table 1).

Ant species composition was studied partially in 2021, and the species identified were *Aphaenogaster senilis* (Mayr), *Tapinoma nigerrimun* (Nylander), *Tetramorium semilaeve* (André), and *Pheidole pallidula* (Nylander), but a thorough identification was carried out in 2022 (Table 2 and Table 3). Ant species in the canopy in 2022 were limited to four and six species in the superintensive and traditional groves, respectively (Table 2). The *Plagiolepis* species (*Plagiolepis pygmaea* (Latreille) and *Plagiolepis schmitzii* Forel) were the most frequent species group (82.7% of the adults identified), especially in the superintensive grove (86.6% of the adults), while, in the traditional grove, they were not as preponderant (44% of the adults). In this latter grove, the diversity of species was higher (with a more relative presence of other species, such as *P. pallidula*, *T. nigerrimun* and *Crematogaster* sp), resulting in diversity indices higher than in the superintensive grove (Appendix B, Table A4), but if the diversity indices obtained with the different sampling dates are compared, the average values were not significantly different (*p* > 0.05, using a paired *t*-test, Appendix B, Table A4) between groves.

Ant species diversity in the pitfall traps in 2022 (Table 3) was higher than in the canopy, with a total of 12 species (eleven different species in superintensive and traditional groves); *P. pallidula* was the predominant species (45.5% of the adults identified), followed by *Plagiolepis* spp. (22.1% of the adults). There were differences between groves regarding the relative importance of the species because, in the superintensive grove, the second and third most abundant species were *Plagiolepis* species (27.7% of the adults) and *A. senilis* (11.2% of the adults), while, in the traditional grove, the second and third most abundant species were *Tetramorium* gr *semilaeve* (18.6% of the adults) and *Plagiolepis* species (8.2% of the adults). The diversity indices obtained in the different combinations of grove and treatment tend to show a higher diversity in the superintensive than traditional grove and more diversity in mineral than compost treatment, especially in the traditional grove (Appendix B, Table A4). However, if the diversity indices obtained with the different sampling dates are compared, the average values were not significantly different (*p* > 0.05, using a paired *t*-test, Table A4) between groves and treatments in any of the comparisons conducted.

The relative importance of the ant species in the canopy in both groves shows statistical differences in four of them. *Plagiolepis* spp. (and particularly *P. smitzii*) were more important in the superintensive grove, whereas *T. nigerrimun* and *P. pallidula* appear more important in the traditional grove (Table 2), indicating differences between both groves. The relative presence of ant species on the ground (Table 3) reflects no significant differences between the two fertilization treatments in both groves and when the type of fertilization is summarized (Table 3), suggesting that the type of fertilization used did not influence species distribution. On the contrary, when summarizing the two groves, some species appear significantly different depending on the grove: *Plagiolepis* spp. (in particular, *P. smitzii*) is again more important in the superintensive grove, together with *Messor barbarus* (Linnaeus), whereas *T.* gr *semilaeve* is more important in the traditional grove.

**Figure 3:**
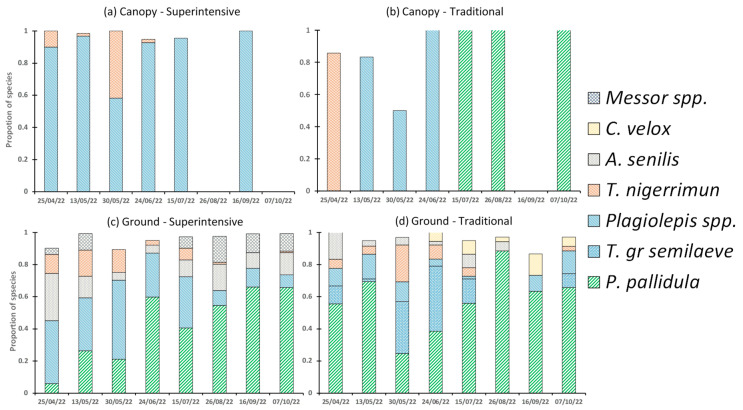
The proportion of ant species on the sampling days of 2022 in the canopy (**a**,**b**) and in the ground (**c**,**d**) in both groves (Superintensive and Traditional).

The species most frequent in the canopy of the superintensive grove in spring 2022 was *Plagiolepis* species (Figure 3a), and in the traditional grove again was *Plagiolepis* spp. the main species, with a secondary presence of *T. nigerrrimun* (Figure 3b). The species’ relative composition in the soil of the superintensive grove showed a shift depending on the timing of the season in 2022: *Plagiolepis* spp. and *A. senilis* were predominant in spring, but *P. pallidula* was predominant in summer and the beginning of autumn (Figure 3c). In the traditional grove, the predominant species was *P. pallidula* during the period of study, followed by *Tetramorium* gr *semilaeve* (only in spring, Figure 3d).

## 4. Discussion

The main objective of the study was to answer the question of whether the addition of ‘alperujo’ compost to olives can affect ants in some way. The answer in this two-year study is that no effect was observed on the numerical response, both at the canopy and the ground level in each grove and in the combination of them. Another important aspect is that the specific composition at ground level was not affected either by the type of fertilization in each grove, although it was determined only in 2022. Ants were captured in greater numbers with the pitfall traps than with the sweeping net, which is normal because ants generally live and dwell on the ground.

A comparison of the two groves with different management (superintensive vs. traditional) produced clear differences between them, but the limited number of replicas (only one grove for each type of crop management) limits the generalization of the conclusion. Although with this limitation, the presence of ants was much more abundant in both the canopy and the soil in the superintensive grove compared to the traditional grove. Different soil management in each grove, especially tillage and cover crops, could be the main factors responsible for these differences by disturbing/destroying ant nests, as different works have stressed [14,18,37,38,45], although other aspects can be confusing, such as fertigation in the superintensive grove, which could increase the presence of phytophagous arthropods in the canopy, particularly in spring, and therefore, the presence of ants and other predators and parasitoids (study under evaluation).

In the traditional grove, tillage is a normal practice (generally conducted in January and July) to avoid (or limit) the proliferation of weeds, whereas, in the superintensive grove, no tillage was applied, it was drip fertigated in the tree rows, the cover crop was permanent, and it was only mowed in the alleys in May–June. Such cultural practices seem to be determinant in drastically reducing ant activity in spring in the traditional grove (in soil and canopy), whereas in the superintensive grove, the ant presence in spring was much greater, it was reduced in summer, when fewer food sources can be found in the canopy but resumed some activity at the end of summer/beginning of autumn. On the contrary, the addition of compost did not produce a significant effect on the ant assemblage, although other arthropods increased its presence, such as Acari, Arachnida, Coleoptera, and other invertebrates (study under evaluation), which agrees with other authors [10,11,12,13].

The ant species composition usually mentioned in different studies in the Iberian Peninsula [29,30,34,36,37,38,41] includes the following (with general decreasing importance): *Cataglyphis* spp., *A. senilis*, *Messor barbarus* (and other *Messor*), *Camponotus* spp., *T. nigerrimum* (and other *Tapinoma*), *P. pallidula*, *Plagiolepis* spp, *T.* gr *semilaeve*, and *Crematogaster* spp. The relative importance of the ant species in our results compares strikingly with the previous list: in the ground survey in both groves, the most frequent species is *P. pallidula*, followed by *Plagiolepis* spp., and the other species are of rather secondary importance. The most frequent species in the canopy was *Plagiolepis* spp. (in both groves again), although in the canopy of the traditional grove other species appear secondarily (*P. pallidula* and *T. nigerrimun*), but in very low quantities. Several works tend to give *T. nigerrimum* (and other *Tapinoma*) an important role in the predation of *P. oleae* larvae (an important pest in olives) in the canopy [35,36,37,46], but the very low presence of this species in the canopy survey of this work does not seem to support a significant role as a predator in both groves, where the presence of *P. oleae* was very low in spring (when anthophagous larvae feed on the olive floral buds and flowers and can be the most accessible prey [47]) in the two years of our study (data under evaluation). However, the ant species highlighted in olive orchards as potential predators of olive pests can have a counter-productive effect on natural enemies [36,37] or boost honeydew producers [48].

The pesticide application was very limited, only to the canopy, and generally in the two groves at the same moments of each season. They were used mainly against *P. oleae* and *B. oleae*, and the low presence of *P. oleae* in buds and flowers can be explained partially by the use of pesticides, but also by the presence along the two seasons of other generalist predators, especially Chrysopidae and spiders (Araneae), together with parasitoids-like hymenopterans in the canopy (study under evaluation), indicating that the application of pesticides did not eradicate arthropods from the canopy.

Species of the genus *Pheidole* move quickly to collect food, such as live or dead insects, food scraps or sugary materials [49]. *Pheidole pallidula* is an omnivorous species, basically a scavenger, although it is also a predator and collects seeds in a smaller proportion than other Formicidae [49], although its interest in predating larva of *P. oleae* and larvae/pupae of *B. oleae* is reduced [37]. In any case, the most frequent species in the canopy (especially in the superintensive grove) were *P. smitzii* and *P. pygmaea*, which appear to be abundant in the olive canopy in some studies [36], and in our survey were by far the most common species in 2022 in the superintensive grove (although the total number of ants in the canopy was very low) and also were the predominant species (or group) in the traditional grove (with even a lower presence), but again their possible interest in predating *P. oleae* larva and *B. oleae* larvae/pupae is reduced [37]. It is also considered an omnivorous species [50] (cited by Morris et al. [36]), with a marked interest in sugary substances [44], and the presence (at low levels) of sap-feeding insects, such as *Euphyllura olivina* Costa (Hemiptera: Psyllidae) nymphs in spring in the superintensive grove that excrete such substances can explain its higher abundance.

Most of the works cited about ants in olive crops in Andalucía (Spain) [29,34,35,36,37,38,46] have been carried out mainly in the provinces of Jaen, Granada, and Córdoba, in areas with characteristics different from where this work has been conducted, and other works were carried out in the north of Portugal [30,31,41], which is also very different. The relative importance of the species found in our work, although conducted only in two groves and for one year (2022), gives the picture of some differences in the ant assemblage depending on the area of survey, even in an aprioristically homogeneous area as the south of Spain could be considered.

## 5. Conclusions

The main conclusion of the present work is that the addition of ‘alperujo’ compost as fertilizer did not produce a significant effect on the quantity and species composition of ants regardless of the grove design and type of management. This result could help to implement the use of ‘alperujo’ compost as a fertilizer in the olive crop, with the main objective of achieving sustainable agriculture. The olive groves (with their distinct characteristics) showed differences in the quantity and assemblages of ant species, with more presence in the superintensive grove than in the traditional grove, probably due to the different soil management in both groves, but the low number of replicas used in this study limits the generalization of this conclusion. The predominant ant species on the ground was *P. pallidula* for both groves, but the secondary species showed differences in each grove. The predominant ant species in the canopy were *Plagiolepis* species for both groves. These conclusions are preliminary, and they should be confirmed with new studies using more groves (replicas) and over a longer period of time.

## Figures and Tables

**Table 1:** Statistics of the analysis conducted each year and grove with the two sampling methods, sweep net and pitfall. The two years and the groves together have also been analyzed.

	2021 ^a^		2022 ^a^		2021–2022 ^b^			
	Superintensive	Traditional	Superintensive	Traditional		Estimate	SE	*p*
**Sweep net**					**Sweep net**			
Treatment (Tr)	*p* = 0.437 [F = 0.74; 1, 4]	- ^c^	*p* = 0.106 [F = 4.3; 1, 4]	*p* = 1 [F = 0; 1, 6]	Fertilization: Mineral/Compost	0.31	0.38	0.415
Sampling date (Sd)	*p <* 0.01 ** [F = 12.1; 2, 8.2]	-	*p <* 0.01 ** [F = 15.3; 1.9, 7.4]	*p* = 0.063 [F = 3.6; 1.9, 11.5]	Grove: Traditional/Superint.	−2.52	0.44	<0.01 **
Tr × Sd	*p* = 0.335 [F = 1.3; 2, 8.2]	-	*p* = 0.191 [F = 2.1; 1.9, 7.4]	*p* = 0.076 [F = 3.3; 1.9, 11.5]	Fertilization × Grove	−0.31	0.62	0.618
**Pitfall**					**Pitfall**			
Treatment (Tr)	*p* = 0.371 [F = 1.01; 1, 4]	*p* = 0.559 [F = 0.383; 1, 6]	*p* = 0.99 [F = 0; 1, 4]	*p* = 0.561 [F = 0.38; 1, 6]	Fertilization: Mineral/Compost	−0.15	0.18	0.416
Sampling date (Sd)	*p* = 0.023 * [F = 9.4; 1.3, 5.3]	*p* = 0.586 [F = 0.42; 1.3, 7.7]	*p* = 0.243 [F = 1.7; 1.8, 7.2]	*p* = 0.154 [F = 2.2; 2.1, 12.6]	Grove: Traditional/Superint.	−1.27	0.18	<0.01 **
Tr × Sd	*p* = 0.150 [F = 2.8; 1.3, 5.3]	*p* = 0.478 [F = 0.66; 1.3, 7.7]	*p* = 0.624 [F = 0.47; 1.8, 7.2]	*p* = 0.868 [F = 0.15; 2.1, 12.6]	Fertilization × Grove	0.14	0.25	0.580

a Statistical analysis conducted with repeated measures ANOVA. The Greenhouse–Geisser’s degree of freedom correction was applied in Sampling date and the interaction Treatment × Sampling date. b Statistical analysis of years and groves together, conducted with a generalized linear model using the binomial negative function. The same number of sampling dates have been used in both groves. c Very few data, and analysis was not carried out. * Significant differences 0.01 < *p* < 0.05, ** Significant differences *p* < 0.01.

**Table 2:** Total numbers and species of ants identified in the two groves (Superintensive and Traditional) in the canopy with the sweep net sampling in 2022; each grove sums the individuals found in compost and mineral fertilization. The numbers between brackets are percentages.

Subfamily	Species	Superintensive	Traditional	Total
Dolichoderinae				
	*Tapinoma nigerrimun*	27 (*10.7*)	6 (*24.0*) (*)	33 *(11.9)*
Formicinae				
	*Plagiolepis* spp.	219 (*86.6*) (**)	11 (*44.0*)	230 (*82.7*)
	*Plagiolepis pygmaea*	85 (*33.6*)	10 (*40.0*)	95 (*34.2*)
	*Plagiolepis smitzii*	134 (*53.0*) (**)	1 (*4.0*)	135 (*48.6*)
Myrmicinae				
	*Crematogaster* sp.	7 (*2.8*)	2 (*8.0*)	9 (*3.2*)
	*Pheidole pallidula*	0	5 (*20.0*) (**)	5 (*1.8*)
	*Tetramorium* gr *semilaeve*	0	1 (*4.0*)	1 (*0.4*)
Total numbers		253	25	278

(*) Significant differences (0.01 < *p* < 0.05) and (**) significant differences (*p* < 0.01), between the pairs of numbers for each species (comparing the frequencies expressed as relative presence with a Χ^2^ test).

**Table 3:** Total numbers and species of ants identified in the two groves (Superintensive and Traditional) and fertilization systems (Mineral and Compost) in the ground with the pitfall traps in 2022; both groves and fertilization treatments are summarized. The numbers between brackets are percentages.

		Superint.	Superint.	Traditional	Traditional	Superintensive	Traditional			
Subfamily	Species	Mineral	Compost	Mineral	Compost			Mineral	Compost	TOTAL
Dolichoderinae										
	*Tapinoma nigerrimun*	46	28	13	18	74 (*7.5*)	31 (*7.9*)	59	46	105 (*7.6*)
Formicinae										
	*Camponotus* sp.	2	0	2	1	2 (*0.2*)	3 (*0.8*)	4	1	5 (*0.4*)
	*Cataglyphis velox*	5	1	13	4	6 (*0.6*)	17(*4.3*)	18	5	23 (*1.7*)
	*Plagiolepis* spp.	151	122	11	21	273 (*27.7*) (**)	32 (*8.2*)	162	143	305 (*22.1*)
	*Plagiolepis pygmaea*	49	36	11	21	85 (*8.6*)	32 (*8.2*)	60	57	117 (*8.5*)
	*Plagiolepis smitzii*	102	86	0	0	188 (*19.1*) (**)	0	102	86	188 (*13.7*)
Myrmicinae										
	*Aphaenogaster senilis*	51	59	11	6	110 (*11.2*)	17 (*4.3*)	62	65	127 (*9.2*)
	*Crematogaster* sp.	1	3	0	2	4 (*0.4*)	2 (*0.5*)	1	5	6 (*0.4*)
	*Messor* spp.	41	30	4	5	71 (*7.2*)	9 (*2.3*)	45	35	80 (*5.8*)
	*Messor* gr *bouveri*	11	7	1	5	18 (*1.8*)	6 (*1.5*)	12	12	24 (*1.7*)
	*Messor* gr *structor*	0	0	2	0	0	2 (*0.5*)	2	0	2 (*0.2*)
	*Messor barbarus*	30	23	1	0	53 (*5.4*) (*)	1 (*0.3*)	31	23	54 (*3.9*)
	*Pheidole pallidula*	221	197	74	134	418 (*42.4*)	208 (*53.1*)	295	331	626 (*45.5*)
	*Tetramorium* gr *semilaeve*	12	15	36	37	27 (*2.7*)	73 (*18.6*) (**)	48	52	100 (*7.3*)
TOTAL NUMBERS		530	455	164	228	985	392	694	683	1377

(*) Significant differences (0.01 < *p* < 0.05) and (**) significant differences (*p* < 0.01), between the pairs of numbers for each species (comparing the frequencies expressed as relative presence with a Χ^2^ test).

## Data Availability

The data presented in this study are available on request from the corresponding author.

## Appendix A

**Table A1:** Fertilization units (kg·ha^−1^) of the principal nutrients applied in the fertilization treatments (Mineral and Compost) in the two groves (Superintensive and Traditional) for the two years of the study.

	Superintensive						Traditional					
	2021						2021					
		Mineral			Compost			Mineral			Compost	
	Foliar	Irrigation	Total	Foliar	Compost	Total	Foliar	Mineral	Total	Foliar	Compost	Total
N	6.4	--	6.4	6.4	14.1	20.5	--	--	--	--	14.1	--
P	--	--	--	--	93.8	93.8	--	--	--	--	93.8	--
K	22.4	--	22.4	22.4	477.0	499.4	--	--	--	--	477.0	--
	2022						2022					
		Mineral			Compost			Mineral			Compost	
	Foliar	Irrigation	Total	Foliar	Compost	Total	Foliar	Mineral	Total	Foliar	Compost	Total
N	6.4	119.0	125.4	6.4	14.1	20.5	8.0	72.9	80.9	8.0	14.1	22.1
P	--	59.5	59.5	--	93.8	93.8	--	24.3	24.3	--	93.8	93.8
K	22.4	178.6	201.0	22.4	477.0	499.4	28.0	97.1	125.1	28.0	477.0	505.0

Olive mill compost used in the superintensive grove: 17 t·ha^−1^ in July 2021 and 17 t·ha^−1^ in March 2022. Olive mill compost in the traditional grove: 17 t·ha^−1^ in December 2020 and 17 t·ha^−1^ in March 2022.

**Table A2:** Treatments against pests, diseases, and weeds in the two groves (Superintensive and Traditional) for the two years of the study.

SUPERINTENSIVE GROVE		
Date	Product used	Used against
1 January 2021	Glyphosate 36%	Mono and Dicotyledoneae ^a^
	MCPA 40%	Dicotyledoneae ^a^
15 February 2021	Copper oxychloride 52%	*Venturia oleaginea*-Other diseases
19 April 2021	Lambda-cyhalothrin 10%	*Prays oleae*
19 May 2021	Deltamethrin 2.5%	*Bactrocera oleae-Prays oleae*
22 June 2021	Glyphosate 36%	Mono and Dicotyledoneae ^a^
	MCPA 40%	Dicotyledoneae ^a^
23 June 2021	Phosmet 50%	*Bactrocera oleae-Prays oleae-Palpita unionalis*
5 November 2021	Copper oxychloride 52%	*Venturia oleaginea*-Other diseases
	Kresoxim methyl 50%	
16 February 2022	Copper oxychloride 52%	*Venturia oleaginea*
20 April 2022	Glyphosate 36%	Mono and Dicotyledoneae ^a^
26 April 2022	Deltamethrin 2.5%	*Prays oleae*
27 May 2022	Kresoxim methyl 50%	*Venturia oleaginea*
	Lambda-cyhalothrin 10%	*Prays oleae*
2 June 2022	Glyphosate 36%	Mono and Dicotyledoneae ^a^
22 June 2022	Sulphur 80%	Eriophyidae
13 July 2022	Phosmet 50%	*Bactrocera oleae-Prays oleae-Palpita unionalis*
4 August 2022	Phosmet 50%	*Bactrocera oleae-Prays oleae-Palpita unionalis*
26 October 2022	Copper oxychloride 52%	*Venturia oleaginea*
TRADITIONAL GROVE		
Date	Product used	Used against
5 November 2021	Copper oxychloride 52%	*Venturia oleaginea*
26 April 2022	Deltamethrin 2.5%	*Prays oleae*
30 May 2022	Deltamethrin 2.5%	*Prays oleae*
	Difenoconazole 23.5%	*Venturia oleaginea*
13 July 2022	Phosmet 50%	*Bactrocera oleae-Prays oleae-Palpita unionalis*
4 August 2022	Phosmet 50%	*Bactrocera oleae-Prays oleae-Palpita unionalis*
27 October 2022	Copper oxychloride 52%	*Venturia oleaginea*

^a^ Herbicides were applied against weeds only in the tree row, where the mower appliance used in the alleys cannot reach.

**Table A3:** Sampling dates in 2021 and 2022 in the two groves (Superintensive and Traditional).

2021			
Superintensive		Traditional	
Sweep net	Pitfall trap	Sweep net	Pitfall trap
12 April 2021	12 April 2021		
3 May 2021	3 May 2021		
14 May 2021			
14 June 2021	14 June 2021	28 June 2021	28 June 2021
12 July 2021	12 July 2021	12 July 2021	12 July 2021
26 July 2021	26 July 2021	26 July 2021	26 July 2021
30 August 2021	30 August 2021	30 August 2021	30 August 2021
20 September 2021	20 September 2021	20 September 2021	20 September 2021
18 October 2021	18 October 2021	18 October 2021	18 October 2021
2022			
Superintensive		Traditional	
Sweep net	Pitfall trap	Sweep net	Pitfall trap
1 April 2022	1 April 2022	1 April 2022	1 April 2022
25 April 2022	25 April 2022	25 April 2022	25 April 2022
13 May 2022	13 May 2022	13 May 2022	13 May 2022
30 May 2022	30 May 2022	30 May 2022	30 May 2022
24 June 2022	24 June 2022	24 June 2022	24 June 2022
15 July 2022	15 July 2022	15 July 2022	15 July 2022
26 August 2022	26 August 2022	26 August 2022	26 August 2022
16 September 2022	16 September 2022	16 September 2022	16 September 2022
7 October 2022	7 October 2022	7 October 2022	7 October 2022

## Appendix B

**Table A4:** Diversity indices used with the ant species found in sweep net and pitfall sampling in two groves (Superintensive and Traditional) and two fertilization treatments (Mineral and Compost) in 2022. The average (standard error between brackets) of the indices in the different sampling dates is also considered.

SWEEP NET								
	Superintensive		Traditional					
Shannon	1.04		1.49					
Simpson	0.59		0.73					
Simpson_Inverse	2.47		3.74					
Simpson_Unbias	0.60		0.76					
	*With sampling dates*							
Shannon	0.34	*(0.16)*	0.29	*(0.11)*				
Simpson	0.19	*(0.10)*	0.19	*(0.07)*				
Simpson_Inverse	1.41	*(0.30)*	1.31	*(0.14)*				
Simpson_Unbias	0.19	*(0.10)*	0.29	*(0.14)*				
PITFALL TRAPS								
	Superintensive				Traditional			
	Mineral		Compost		Mineral		Compost	
Shannon	1.75		1.69		1.63		1.34	
Simpson	0.76		0.75		0.73		0.61	
Simpson_Inverse	4.16		3.94 0.75		3.65		2.58 0.61	
Simpson_Unbias	0.76				0.73			
			*With sampling dates*					
Shannon	1.25	*(0.10)*	1.30	*(0.06)*	1.16	*(0.13)*	0.98	*(0.17)*
Simpson	0.63	*(0.05)*	0.65	*(0.03)*	0.59	*(0.05)*	0.49	*(0.09)*
Simpson_Inverse	2.96	*(0.35)*	3.03	*(0.28)*	2.84	*(0.47)*	2.38	*(0.38)*
Simpson_Unbias	0.64	*(0.05)*	0.67	*(0.03)*	0.63	*(0.05)*	0.52	*(0.09)*
	Superintensive		Traditional					
Shannon	1.73		1.50					
Simpson	0.75		0.67					
Simpson_Inverse	4.06		3.00					
Simpson_Unbias	0.75		0.67					
	*With sampling dates*							
Shannon	1.34	*(0.08)*	1.17	*(0.12)*				
Simpson	0.65	*(0.03)*	0.56	*(0.06)*				
Simpson_Inverse	3.07	*(0.29)*	2.56	*(0.31)*				
Simpson_Unbias	0.66	*(0.03)*	0.58	*(0.06)*				
	Mineral		Compost					
Shannon	1.83		1.69					
Simpson	0.77		0.72					
Simpson_Inverse	4.30		3.59					
Simpson_Unbias	0.77		0.72					
	*With sampling dates*							
Shannon	1.42	*(0.11)*	1.37	*(0.09)*				
Simpson	0.67	*(0.05)*	0.65	*(0.04)*				
Simpson_Inverse	3.40	*(0.42)*	3.18	*(0.41)*				
Simpson_Unbias	0.67	*(0.05)*	0.66	*(0.04)*				

A paired *t*-test was used to test the significance (*p* < 0.05) of the diversity indices obtained with the sampling dates, but none of the comparations were significant.
